# Biochemical and genetic predictors of overall survival in patients with metastatic pancreatic cancer treated with capecitabine and nab-paclitaxel

**DOI:** 10.1038/s41598-017-04743-0

**Published:** 2017-07-07

**Authors:** Daniela Bianconi, Gerwin Heller, Daniel Spies, Merima Herac, Andreas Gleiss, Sandra Liebmann-Reindl, Matthias Unseld, Markus Kieler, Werner Scheithauer, Berthold Streubel, Christoph C. Zielinski, Gerald W. Prager

**Affiliations:** 1 Department of Medicine I, Comprehensive Cancer Center Vienna, Medical University of Vienna, Vienna, Austria; 2 Swiss Federal Institute of Technology Zurich, Department of Biology, Institute of Molecular Health Sciences, Otto-Stern Weg 7, 8093 Zurich, Switzerland; 3 Life Science Zurich Graduate School, Molecular Life Science Program, Institute of Molecular Life Science, University of Zurich, Winterthurerstrasse 190, Zurich, 8057 Switzerland Austria; 4 Clinical Institute of Pathology, Medical University of Vienna, Vienna, Austria; 5 Center for Medical Statistics, Informatics, and Intelligent Systems, Medical University of Vienna, Vienna, Austria; 6 Core facilities, Medical University of Vienna, Vienna, Austria

## Abstract

Pancreatic cancer is a dismal disease with a mortality rate almost similar to its incidence rate. To date, there are neither validated predictive nor prognostic biomarkers for this lethal disease. Thus, the aim of the present study was to retrospectively investigate the capability of biochemical parameters and molecular profiles to predict survival of patients with metastatic pancreatic ductal adenocarcinoma (mPDAC) who participated in a phase II clinical trial to test the safety and efficacy of the combination treatment of capecitabine plus nab-paclitaxel. Herein, we investigated the association of 18 biochemical parameters obtained from routine diagnosis and the clinical outcome of the 30 patients enrolled in the clinical trial. Furthermore, we analysed formalin-fixed paraffin-embedded (FFPE) tumour tissue to identify molecular biomarkers via RNA seq and the Illumina TruSeq Amplicon Cancer panel which covers 48 hotspot genes. Our analysis identified SERPINB7 as a novel transcript and a DNA mutation signature that might predict a poor outcome of disease. Moreover, we identified the bilirubin basal level as an independent predictive factor for overall survival in our study cohort.

## Introduction

One of the most common disorders associated with the small but multi-tasking pancreas is pancreatic cancer^1^; a disease which has a very dismal prognosis and a significant impact in the quality of life of patients. In 2012, pancreatic cancer was reported as the seventh most common cause of cancer-related deaths worldwide and according to the Pancreatic Cancer Action Network, it will be the second one by the year 2030^2, 3^. This discouraging prognosis is partly due to the absence of reliable biomarkers and the insufficient understanding of the molecular mechanisms underlying the pathology of pancreatic cancer.

For many years, gemcitabine was the only drug approved to treat this malignant disease. Since then, one of the most significant advances was the FDA approval of nab-paclitaxel (an albumin-bound paclitaxel) to treat metastatic pancreatic cancer based on the results of the MPACT study^4^. In 2013, this phase III clinical trial demonstrated that patients treated with nab-paclitaxel and gemcitabine had a prolonged overall survival (OS) and progression free survival (PFS) in comparison to patients treated with gemcitabine alone^4^. These results have prompted several studies exploring nab-paclitaxel in combination with other therapeutic agents to treat pancreatic cancer. However, despite the efficacy of nab-paclitaxel found in different types of cancer, the precise mechanisms of action are yet unknown. Unlike paclitaxel, nab-paclitaxel is a solvent-free formulation that does not form micelles upon systemic exposure, which might explain to some extent the higher uptake by the tumour in comparison to paclitaxel^5^. Further on, evidence suggests that the endothelial cell albumin-surface receptor gp60 might mediate transcytosis of the nanoparticle, contributing to an intratumoral accumulation of the agent^6^. Nevertheless, up to now, there is no predictive biomarker for nab-paclitaxel responsiveness. One potential candidate was the secreted protein acidic and rich in cysteine (SPARC; also known as osteonectin or BM-40) which binds albumin and is overexpressed in the tumour stroma^7^. However, meta-analysis revealed that tissue SPARC expression did not predict nab-paclitaxel efficacy^8^. Overall, this data evinces that there is a need for new insights into the pathogenesis of pancreatic cancer and putative biomarkers.

Herein, we sought to analyse post-hoc a small but well defined cohort of patients who enrolled in a monocentric academic phase II clinical trial Met.Panc.01 (EudraCT 2013-001714-15) which was initiated to establish safety and efficacy of the combination of capecitabine with nab-paclitaxel in patients with previously untreated metastatic pancreatic ductal adenocarcinoma (mPDAC)^9^. The main aim of the present study was to identify predictors of clinical outcome that might help stratify patients who might benefit from this chemotherapeutic treatment. For this purpose, we analysed differential gene expression as well as differences in mutational signatures between short and long surviving patients with metastatic pancreatic cancer using next-generation sequencing (NGS) approaches.

## Results

### Baseline bilirubin level might be a predictor of overall survival in mPDAC

The basic characteristics of the 30 patients enrolled in this clinical study are summarized in Table 1. Ninety percent of the patients had an ECOG performance status of 1 and 47% were females^10^. All of the patients had metastasis and most of them were found in the liver (80%). In six cases (20%), the primary tumour was located in the tail of the pancreas. Nine patients (30%) had the primary tumour in the corpus and the remaining patients (50%) had the primary tumour in the head +/− corpus of the pancreas. The primary tumour was removed in two cases (7%) and seven patients (23%) had a biliary stent at trial entry. The median follow-up of the present updated analysis was 24.6 months (quartiles: 18.4–26.2 months). The median OS was 10.2 months and the median PFS was 5.2 months. At the time of analysis, 5 patients were still alive.

**Table 1:** Baseline characteristics of participants (n = 30).

Characteristics	n [%]
***Age***	
Median, years [range]	64.5 [37–80]
≥65 years	14 [47]
***Sex***	
Female	14 [47]
***ECOG PS***	
0	27 [90]
1	3 [10]
**Site(s) of metastasis**	
Lung	3 [10]
Liver	24 [80]
Peritoneum	3 [10]

Dose reduction was required in eight cases^9^. Neither the location or removal of the primary tumour nor the presence of biliary stents showed a significant influence on the dose reductions applied in this study (p = 0.457, p = 0.08 and p = 1.00, respectively). The log-rank test was used to determine if the three characteristics mentioned above affected OS. Location and removal of the primary tumor and the presence of biliary stents failed to reach statistical significance (p = 0.595, p = 0.390 and p = 0.226, respectively).

Because nab-paclitaxel showed more efficacy than the solvent-based paclitaxel and is found in an unbound form in the blood, we hypothesized that some biochemical parameters might interfere with the efficacy of nab-paclitaxel. Thus, the Cox proportional hazards model was used to evaluate the association of OS and PFS with each of 16 biochemical parameters and two blood tumour markers (CA19-9 and CEA) in a univariate analysis (Table 2). None of the parameters exhibited a statistically significant influence on PFS. However, the bilirubin level showed a statistically significant influence on OS (Table 2). The instantaneous risk (hazard) of death increases by 77% on average (95% confidence interval: 23% to 154%, adjusted p-value = 0.038) with every doubling of the bilirubin value.

**Table 2:** Cox model analysis for biochemical parameters and OS and PFS.

Parameter	OS				PFS			
	HR	95% CI	Unadj. p-value	Adj. p-value	HR	95% CI	Unadj. p-value	Adj. p-value
Albumin	1,04	0,98–1,10	0,208	1,000	1,02	0,98–1,07	0,315	1,00
Chloride^#^	0,97	0,34–2,82	0,961	1,000	0,91	0,37–2,24	0,838	1,00
Cholesterol^+^	0,76	0,51–1,14	0,049	0,830	1,12	0,70–1,80	0,640	1,00
Cholinesterase	0,85	0,66–1,10	0,212	1,000	0,97	0,77–1,22	0,788	1,00
Fibrinogen Clauss^§^	1,11	0,73–1,66	0,632	1,000	1,14	0,78–1,68	0,493	1,00
Calcium^°	0,88	0,62–1,26	0,483	1,000	1,05	0,76–1,45	0,772	1,00
Total protein	1,01	0,91–1,13	0,840	1,000	1,04	0,96–1,12	0,359	1,00
Alanine transaminase (ALT)*	1,14	0,82–1,58	0,431	1,000	1,31	0,96–1,79	0,088	1,00
Alkaline phosphatase*	0,94	0,57–1,53	0,791	1,000	0,85	0,53–1,37	0,503	1,00
Aspartate transaminase (AST)*	1,33	0,79–2,25	0,282	1,000	1,22	0,76–1,97	0,410	1,00
Bilirubin*	1,77	1,23–2,54	0,002	0,038	1,29	0,93–1,80	0,130	1,00
Carbohydrate antigen 19-9 (CA19-9)*	1,07	0,98–1,17	0,155	1,000	1,07	0,98–1,17	0,156	1,00
Carcinoembryonic antigen (CEA)*	0,98	0,85–1,13	0,735	1,000	0,99	0,85–1,14	0,843	1,00
Creatine kinase (CK)*	1,02	0,56–1,85	0,955	1,000	0,81	0,48–1,36	0,426	1,00
C-reactive protein (CRP)*	1,12	0,88–1,41	0,359	1,000	1,13	0,90–1,40	0,289	1,00
γ-glutamyltransferase (GAMMAGT)*	1,00	0,78–1,28	0,985	1,000	1,06	0,85–1,32	0,611	1,00
Creatinine*	4,03	0,73–22,20	0,110	1,000	2,30	0,46–11,44	0,308	1,00
Lactate dehydrogenase (LDH)*	0,52	0,14–1,96	0,332	1,000	0,48	0,15–1,52	0,213	1,00

HR refers to the hazard ratio. 95% CI refers to 95% confidence interval. Unadj. = unadjusted, Adj. = adjusted. ^#^HR refers to the effect of a 10 mmol/l increase. ^+^Significantly time-dependent effect, reported HR is for OS = 10 months, reported p-value refers to total effect. ^§^HR refers to the effect of a 100 mg/dl increase. ^°HR refers to the effect of a 0.1 mmol/l ncrease. ^*^Calculated with log2-transformed parameter, HR refers to effect of doubling of parameter value.

### High expression of SERPINB7 might predict poor outcome in mPDAC

**Figure 1:**
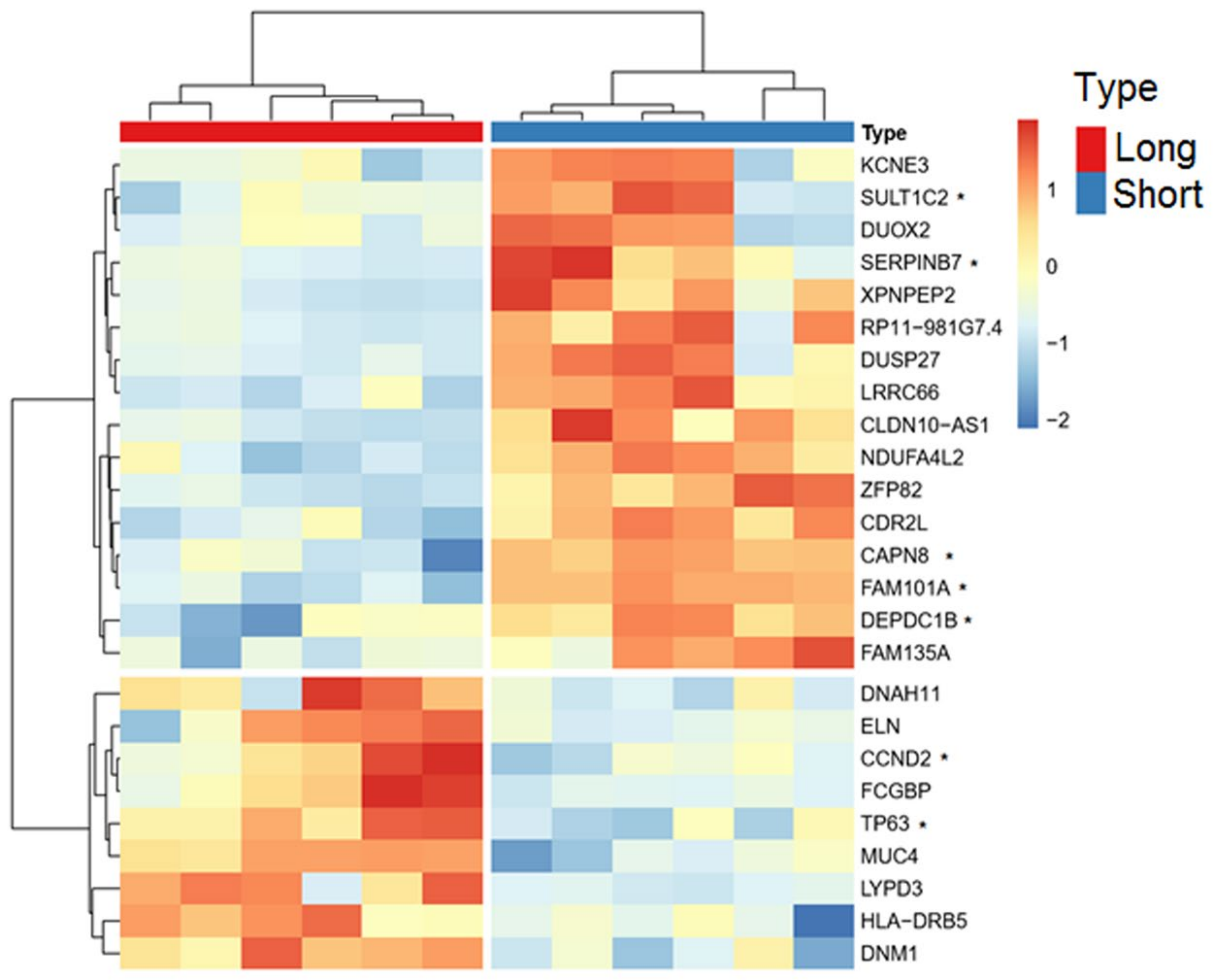
Heat map depicting relative transcript levels of differentially expressed genes in patients with metastatic pancreatic cancer (short vs. long survivors). Lower and higher expression are depicted in blue and red, respectively. Each row indicates a single gene and each column indicates a clinical sample. Each patient was analysed in duplicate. Sample identifiers have been removed. Asterisks indicate the genes which remain significant in the univariate analysis of the validation cohort (non adj. p-value < 0.05).

The transcriptomes of six pancreatic cancer patients with liver metastasis were analysed by RNA-seq. The median OS of these six patients was 13.5 months and was used as a cut-off to distinguish between long and short survivors. RNA-seq data from the long survivors were compared to the short survivors using two different algorithms (DESeq and EdgeR). The common changes in transcript expression between both groups are depicted in Fig. 1. (The results of the individual algorithms can be found in the Supplemental Material S4). 25 transcripts were found to be differentially expressed. 16 genes had a lower expression in the group of the long survivors and nine transcripts had a lower expression in the short survivor group. GO enrichment analyses^11^ revealed that these genes are not involved in common molecular pathways. In addition, RNA-seq data were used to detect fusion transcripts, however, no fusion transcripts were found in any clinical sample analysed.

**Figure 2:**
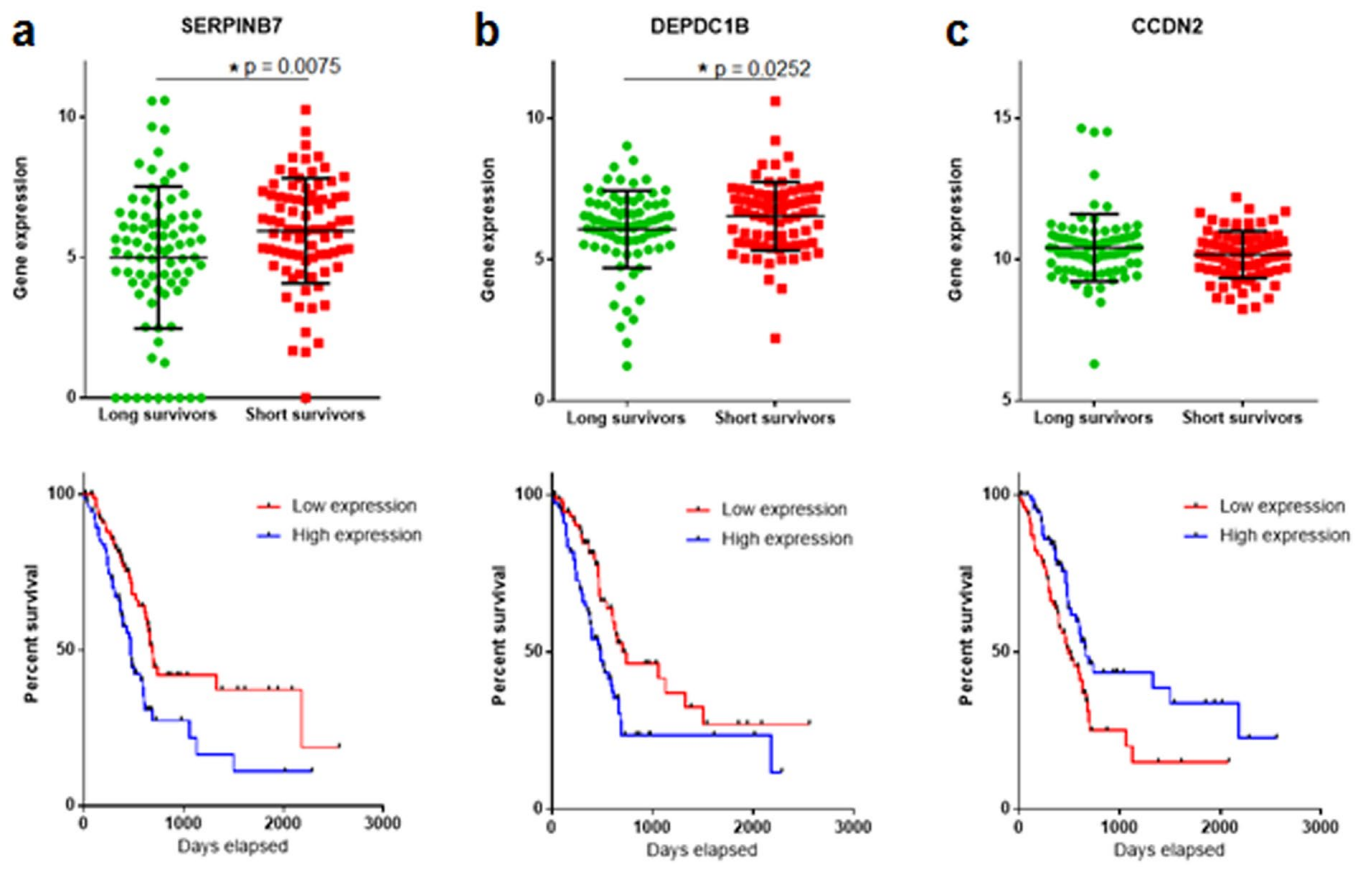
Validation of genes differentially expressed in the short and long survivor groups. Gene expression level of SERPINB7 (**A**), DEPDC1B (**B**) and CCDN2 (**C**) in the short and long survivor groups. P values were calculated using the unpaired t-test. At the bottom, Kaplan-Meier curves for OS based on the expression level of SERPINB7 (**A**), DEPDC1B (**B**) and CCDN2 (**C**).

These findings were then validated using an independent dataset of The Cancer Genome Atlas (TCGA) dataset^12^. For this purpose, samples of the TCGA were divided into two groups according to the expression level of each of the genes listed in Fig. 1. Samples with a gene expression level above the median expression value were classified as “high expression” and samples below the median value were classified as “low expression”. First, the association of the OS and gene expression level was assessed by Kaplan-Meier curves (log-rank test) to select candidates for the multivariate analysis. Univariate analyses revealed that the expression level of CAPN8 (unadj. p = 0.0288), CCND2 (unadj. p = 0.0091), DEPDC1B (unadj. p = 0.0055), FAM101A, (unadj. p = 0.0273), SERPINB7 (unadj. p = 0.0031), SULT1C2 (unadj. p = 0.0464) and TP63 (unadj. p = 0.0283) correlated with OS in the TCGA cohort. (These genes are marked with an asterisk in Fig. 1). SERPINB7, DEPDC1B and CCND2 remained significant after false-discovery rate adjustment (FDR < 0.1) (p = 0.06875; p = 0.06875 and p = 0.07583333, respectively). We then used the Cox’s regression model (including age and stage) to determine if the expression level of these genes were independent predictors of OS in the multivariate analysis. The Cox model revealed that the expression level of SERPINB7 was an independent predictor of OS (HR = 1.189, 95% CI: 1.048 to 1.350, p = 0.007). The Kaplan-Meier curve for OS based on the gene expression level of SERPINB7 is depicted in Fig. 2A (at the bottom). SERPINB7 expression was found to be higher in the short survivor group compared to long survivors (Fig. 2A, at the top). According to the unpaired t-test, this difference was significant (p = 0.0075). Of note, the expression level of DEPDC1B and CCDN2 were found to be independent predictors of OS when individual p-values were analysed in the multivariate analysis according to the likelihood ratio test (p = 0.0047 and p = 0.00889), the Wald test (p = 0.0212 and p = 0.0278) and the log-rank test (p = 0.016 and p = 0.0223, respectively) (Fig. 2B and C).

### Increased mutational load might predict poor prognosis

To identify DNA mutations that might predict better outcome in our study cohort, we used the TruSeq Amplicon Cancer Panel (TSACP) from Illumina which includes 48 cancer-related genes. Analyses were performed on 12 FFPE tumour samples. Two samples were obtained from the primary tumours and ten samples were obtained from metastatic sites (eight cases from the liver and two cases from the lung).

**Figure 3:**
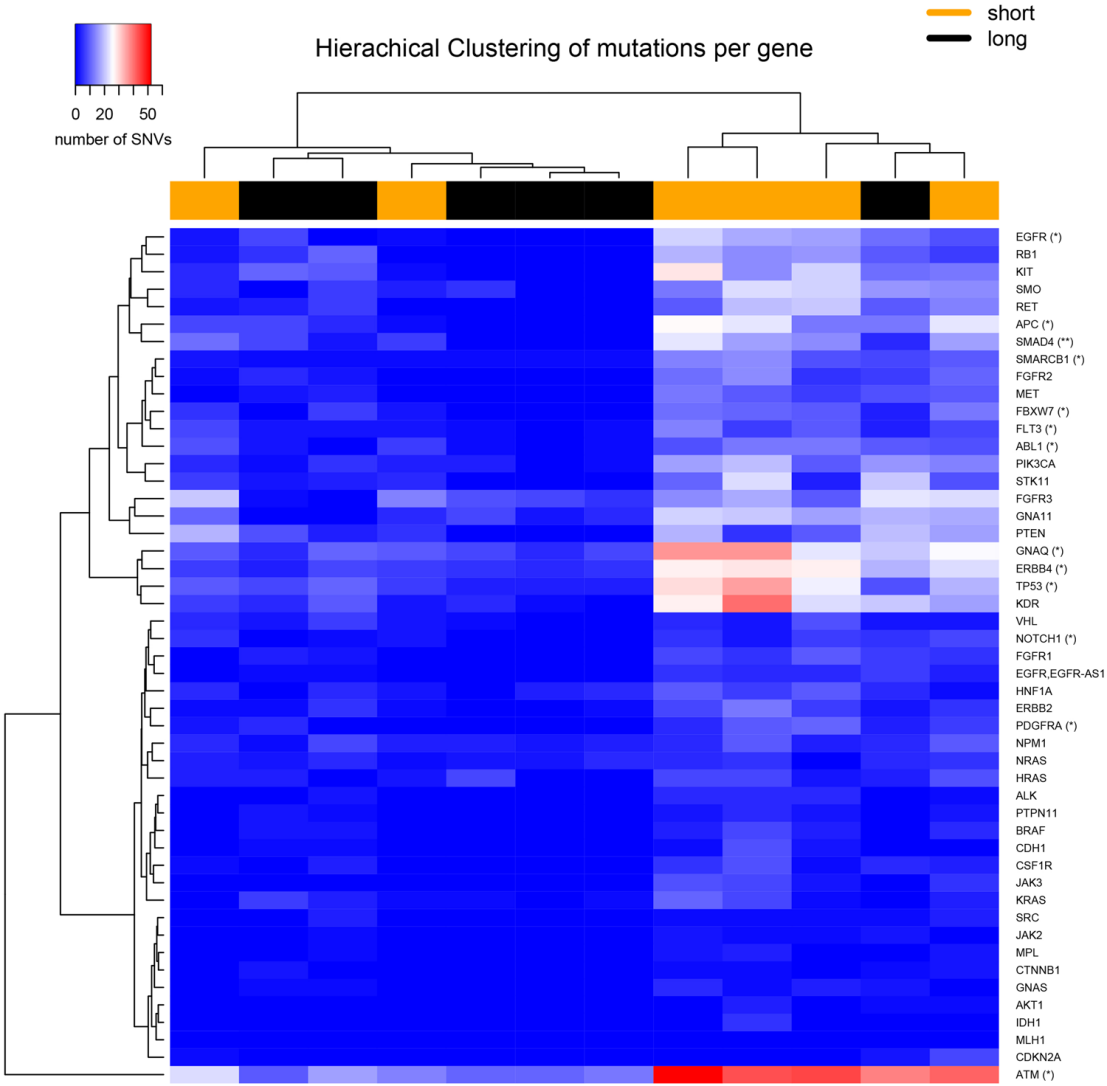
Heatmap of DNA mutations between short and long survivors in the cohort study. Mutations were summed up for each gene and hierarchical clustering performed. Significant genes of a Wilcox ranked sum test between long and short survival patients are indicated by having a p-value of (*) 0.05 and (**) 0.01 (not adjusted).

Hierarchical cluster analysis revealed that there was no difference between the origin of tissue and specific single nucleotide variations (SNV). However, samples clustered according to the OS (short survivors <median OS and long survivors >median OS), whereas short survivors exhibited an increased mutational load (Fig. 3). A Wilcox ranked sum test between short and long survivor groups reported EGFR, APC, SMAD4, SMARCB1, FBXW7, FLT3, ABL1, GNAQ, ERBB4, TP53, NOTCH1, PDGFRA and ATM as top ranking genes associated with a poor prognosis (Supplemental Material S5).

### One mutational signature predicts poor OS in mPDAC

**Figure 4:**
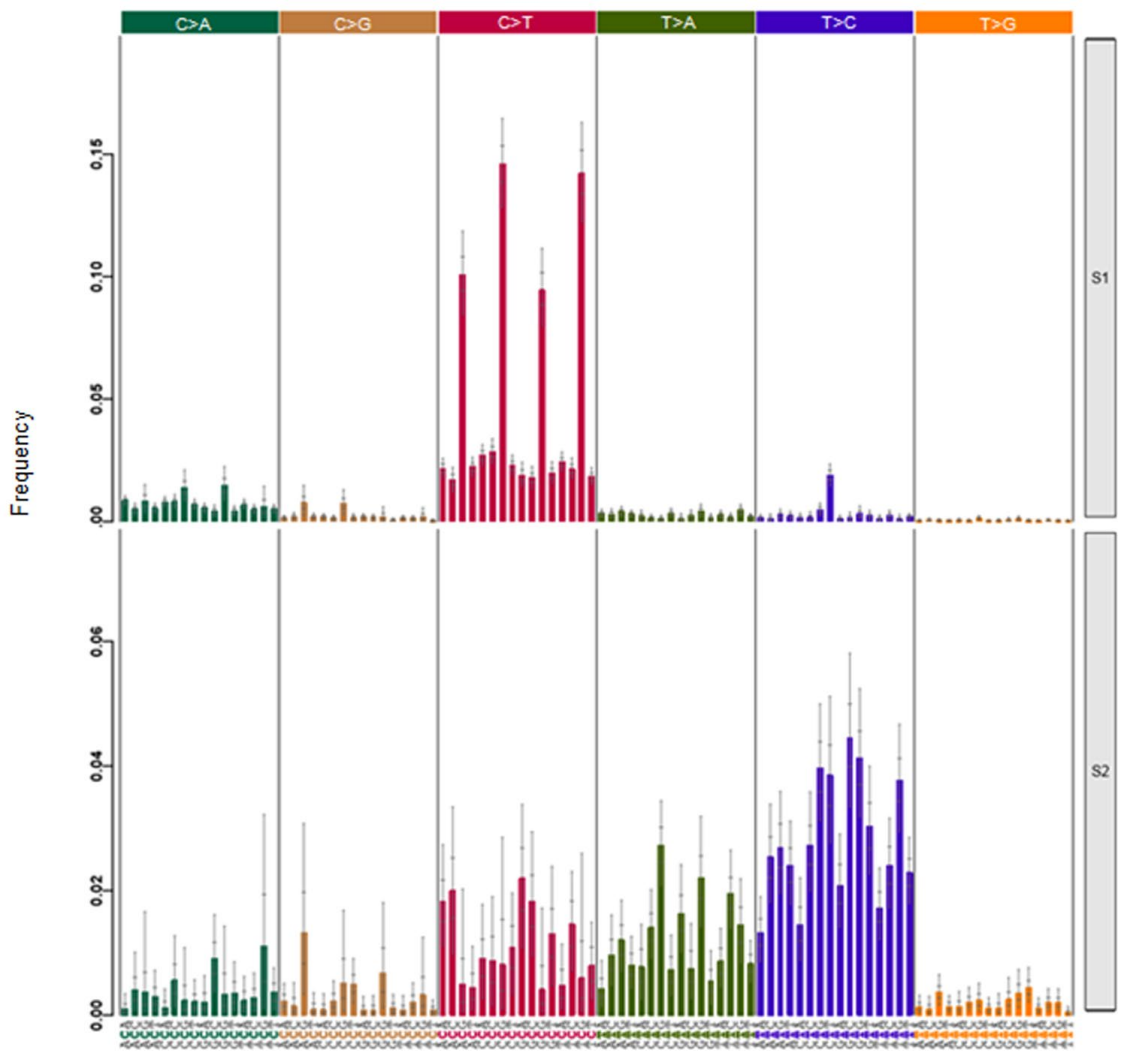
Patterns of the two mutational signatures (S1 and S2) identified in the study cohort. X axes exhibit the 16 mutation types as described in ref. 24.

**Figure 5:**
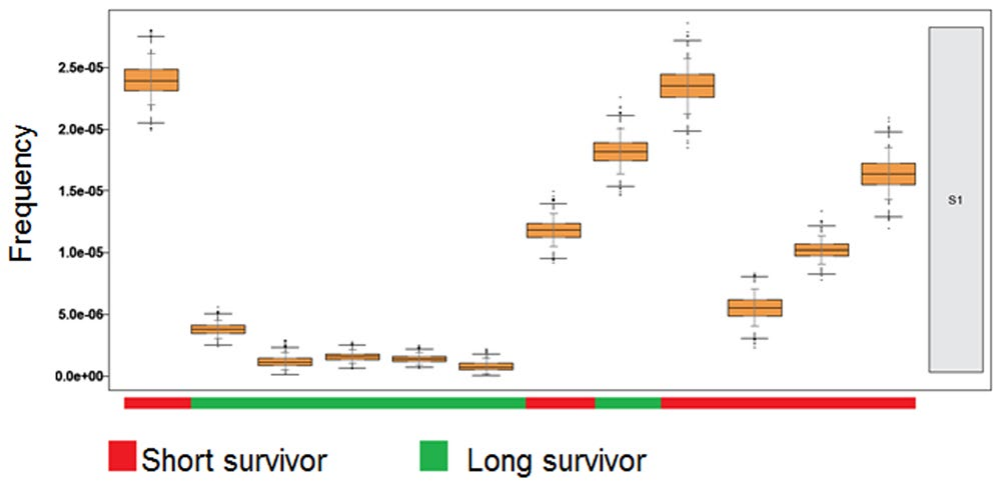
Association of the mutational signature S1 with short overall survival. Contribution of each sample to the mutational signature S1. Sample identifiers have been removed.

Mutational signatures were analysed in the tumour tissue to determine if specific signatures could help predict treatment response. Two distinct mutational signatures were identified. While signature S1 exhibited principally C > T substitutions (Fig. 4A), signature S2 exhibited mostly T > C substitutions and to a lesser extent, C > T and T > A substitutions (Fig. 4B). We then investigated if one of these mutational signatures was associated with OS of the patients. As shown in Fig. 5, signature S1 was predominantly found in the short survivor group and this difference was found to be statistically significant as compared to long survivors (p = 0.02497).

## Discussion

It was not until 2000 that the first progression model for pancreatic cancer was proposed^13^. At that time, it was already known that the most common mutated gene in pancreatic cancer was the KRAS oncogene and that other common mutations were mutations affected the genes *TP53, DPC4*, and *BRCA2*, which appear later than the *KRAS* mutation^13, 14^. Thanks to the technological advances such as NGS, new putative cancer drivers and candidates for targeted therapy were recently proposed and insights into the pathogenesis of pancreatic cancer were gained^15–20^. Notwithstanding of these recent advances, there are still neither prognostic nor predictive biomarkers for this devastating disease and thus, the present retrospective study assessed predictive biomarkers for patients with pancreatic cancer treated with capecitabine plus nab-paclitaxel.

In the first place, we investigated the utility of the biochemical parameters usually assessed in the clinical routine to predict outcome of disease. We found that the higher the level of bilirubin, the higher the risk of death is, even if the bilirubin levels were within normal values. On the one side, nab-paclitaxel is a 130 nm albumin-bound paclitaxel formulation, which anti-tumour efficacy is driven by the unbound form upon systemic exposure^5^. On the other side, serum albumin functions as a carrier for bilirubin in the blood system and facilitates its uptake by the hepatocytes in the liver^21^. These two facts led us to hypothesize that the association found between the bilirubin level and risk of death might be due to interactions in the blood system between nab-paclitaxel and bilirubin. In other words, the higher the level of bilirubin, the lower the proportion of unbound nab-paclitaxel found in bloodstream and, therefore, the lower its efficacy.

Further, in this study we analysed the transcriptome of liver metastatic sites of short and long survivors and validated our results using an independent RNA-seq dataset of the TCGA database. We found that low expression of SERPINB7 predicted a prolonged overall survival in our cohort study as well as in the validation cohort. Previous research has demonstrated that SERPINB7 was overexpressed in lung and breast cancer tissue and that its overexpression inhibited cell migration and cell invasion *in vitro* ^22^. Conversely, SERPINB7 was found to be downregulated in oral squamous cell carcinoma^23^. Currently, no evidence has yet been provided for the functionality, role or expression of SERPINB7 in the pancreas or pancreatic cancer and therefore, it is difficult to speculate about a plausible mechanism whereby expression of SERPINB7 could affect treatment response. Noteworthy, there is a meaningful difference between our study cohort and the validation cohort. The TCGA cohort used primary tumour tissue to perform RNA-seq analysis, while we used metastatic tumour tissue. Thus, this might indicate that expression of SERPINB7 might predict OS, independently of the origin of tissue. Another important issue of consideration is that the patients of the TCGA cohort did not receive the same treatment as our patients and therefore, this could also indicate that the expression of SERPINB7 might be prognostic (and not predictive). Taken this together, it is clear that our findings need to be validated in an independent RNA-seq dataset based on liver metastatic tumour tissue from patients treated with capecitabine and nab-paclitaxel.

We then focussed on the question of whether DNA mutations could help identify patients that are more likely to benefit from the treatment regimen presented herein or not. Therefore, we analysed DNA mutations in 48 genes using cancer panels. Although we did not identify specific gene mutations that enable stratification of patients, we observed that an increased mutational load correlated with a shorter OS. Moreover, we identified two mutational signatures in our study cohort. The mutational profile of signature S1 was similar to the signature 1A reported previously by Alexandrov *et al*., which might be attributable to the spontaneous deamination of 5-methyl-cytosine^24^. Signature S2 was similar to the signature 12 reported before, which was found predominantly in hepatocellular carcinoma^24^. Herein, we reported for the first time that the mutational signature S1 predicts a poor outcome in patients with mPDAC treated with capecitabine and nab-paclitaxel.

### Conclusions and limitations of the study

To the best of our knowledge, this is the first study that identifies potential predictive biomarkers of metastatic pancreatic cancer using NGS. This is especially important since almost all of the patients present one or more metastatic sites at diagnosis and therefore, this applies to most of the clinical situations. Moreover, we did not only investigate mutations at the DNA level or biochemical parameters that might affect therapy efficacy but also the transcriptome of patients with mPDAC. The discouraging results from the vast majority of studies focussed on pancreatic cancer might suggest that the gene dysregulation in PDAC might be regulated by other mechanisms, such as epigenetic or post-transcriptional regulation, and therefore we are convinced that finding RNA biomarkers for PDAC could be extremely useful to provide a deeper understanding of PDAC and to advance therapeutic strategies.

Although the results presented herein are very promising, this study contains three main limitations. First, the reader should keep in mind that the findings reported in this study are only true for the combination of the two chemotherapeutic agents capecitabine and nab-paclitaxel. The second limitation is the small sample size. The small cohort of patients may lead to false negative results due to low statistical power. For instance, this means that we cannot discard the possibility that the level of some biochemical parameters do correlate with OS or PFS. The third limitation is the material used for the NGS experiments. It is known that DNA and RNA isolated from FFPE samples are more fragmented and are chemically modified than those derived from frozen tissue; however, different studies demonstrated that FFPE material is a robust source for NGS^25–27^. Overall, the results of this study provide new information about biomarkers that might predict clinical outcome in patients with metastatic pancreatic cancer treated with nab-paclitaxel; however, our findings need to be validated in future studies.

## Methods

### Study Design

This study is based on a phase II clinical trial^9^. The study was conducted in accordance with the ethical principles outlined in the Declaration of Helsinki and the study protocol was approved by the local ethical committee of the Medical University of Vienna (EudraCT 2013-001714-15). Patients enrolled in this study signed an informed consent form. For the RNA and DNA analysis, we used the maximum number of samples possible (Supplemental Material S1). The remaining samples were insufficient to perform the experiments. All data generated or analysed during this study are included in this article (and its Supplementary Information files).

### RNA Sequencing

Six patient samples were chosen for RNA-seq analyses. The criteria for this selection were: (1) same tissue origin, enough material and possible cut-off between long and short survivals. Total RNA was isolated from tumour cells of formalin fixed paraffin embedded (FFPE) tissue sections using the RNeasy FFPE Kit (Qiagen) according to manufacturer’s instructions. Each sample was processed in duplicates. RNA quality control was performed using the Bioanalyzer RNA 6000 Pico (Agilent). RNA-seq libraries were prepared using SMARTer Universal Low Input RNA Kit for Sequencing (Clonthech) according to manufacturer’s instructions. Sequencing was performed using the Illumina HiSeq 3000 Sequencing System (75 bp paired end sequencing). Raw RNA-seq data were aligned to hg19 using TopHat2 algorithm^28^. Differentially expressed transcripts were detected using DESeq2 (version 1.14.1) and EdgeR (version 3.16.5) algorithms^29, 30^. The presence of fusion transcripts was analysed using FusionCatcher^31^.

### DNA gene panel sequencing

Genomic DNA was isolated from formalin-fixed, paraffin-embedded (FFPE) tumour tissue sections using QiaAmp DNA FFPE Tissue Kit (Qiagen) according to the manual provided by the manufacturer. DNA quantification was conducted using Qubit® dsDNA HS Assay Kit (Invitrogen). Selected specimens were subjected to library preparation for Next-Generation Sequencing (NGS) using Illumina TruSeq Amplicon Cancer panel, which allows parallel investigation of 48 cancer-relevant genes (Supplemental Material S2). Subsequent library preparation was conducted with TruSeq Amplicon Cancer Panel according to the reference guide provided by Illumina Inc. Pooled Libraries were sequenced on the MiniSeq Illumina platform using MiniSeq High Output (300 cycles) sequencing chemistry.

Primary data analysis was performed using the TruSeq Amplicon workflow (Version 2.0.0.0.) via Basespace Sequence Hub. (Alignment to hg19 reference genome with SAMtools Isis Smith-Waterman-Gotoh and base calling with somatic variant caller) For primary filtering and annotation of all datasets Illumina Variantstudio v2.2 software was used. LowGQ and synonymous variants were filtered before single nucleotide variants (SNVs) were counted for each gene separately. Gene SNV counts were then hierarchically clustered using the heatmap.2 function of the gplots R package. Mutation signatures were calculated and visualized using the R package signer (version 1.0.1)^32^.

### Publicly available datasets

RNA-seq data and clinical data from the PAAD TCGA dataset were obtained from https://genome-cancer.ucsc.edu/ (Supplemental Material S3). Overall, data from 163 patients were available for analyses.

### Statistical analysis

The distribution of age is described as median and range, categorical variables are described as counts and percentages. Median OS and median PFS are deduced from Kaplan-Meier survival estimates^33^. Median follow-up length is calculated using the inverse Kaplan-Meier method. The potential influence of each biochemical parameter on OS and PFS was investigated using separate univariate Cox proportional hazard regression models^33^. Hazard ratios (HR) and 95% confidence intervals (CI) are reported. P-values are adjusted for testing multiple parameters using the method of Bonferroni-Holm^34^. Right-skewed parameters were log2-transformed before used in the model; hazard ratios for these parameters thus quantify the effect of each doubling of the parameter value. Each parameter was tested for non-linear (i.e. cubic, quadratic) and time-dependent (i.e. interaction with log of survival time) effects. No significant non-linearities were detected. In case of time-dependence the hazard ratio (HR) is reported for a pre-specified survival time.

Descriptive statistics and Cox regression were done using SAS 9.4. (SAS Institute Inc., 2012). P-values ≤ 0.05 are considered statistically significant.

Possible influences of SNV in single genes on the survival was assessed by a Wilcoxon rank sum test, comparing short and long survival groups using the standard Wilcox.test function in R. Multiple test correction was performed using the “Benjamini & Hochberg” method of the standard R p.adjust function (version 3.0.1)^35, 36^.

## Electronic supplementary material

Supplemental Information
