# Supplementary material for: Biochemical and genetic predictors of overall survival in patients with metastatic pancreatic cancer treated with capecitabine and nab-paclitaxel
Source: Sci Rep. 2017 Jul 7;7:4851. doi: 10.1038/s41598-017-04743-0 (PMC5501799; doi:10.1038/s41598-017-04743-0)
Supplement: Supplementary file 1 — Supplemental Information [file 41598_2017_4743_MOESM1_ESM.pdf]

Biochemical and genetic predictors of overall survival in patients with metastatic pancreatic cancer treated with capecitabine and nab-paclitaxel

Daniela Bianconi[1], Gerwin Heller[1], Daniel Spies[2], Merima Herac[3], Andreas Gleiss[4], Sandra Liebmann-Reindl[5], Matthias Unseld[1], Markus Kieler[1], Werner Scheithauer[1], Berthold Streubel[3], Christoph C. Zielinski[1], Gerald W. Prager[1*]

[1]Department of Medicine I, Comprehensive Cancer Center Vienna, Medical University of Vienna, Austria.

[2]Swiss Federal Institute of Technology Zurich, Department of Biology, Institute of Molecular Health Sciences, Zurich, Otto-Stern Weg 7, 8093 Zurich, Switzerland ; Life Science Zurich Graduate School, Molecular Life Science Program, University of Zurich, Institute of Molecular Life Sciences, Winterthurerstrasse 190, 8057 Zurich, Switzerland.

[3]Clinical Institute of Pathology, Medical University of Vienna, Vienna, Austria.

[4] Center for Medical Statistics, Informatics, and Intelligent Systems, Medical University of Vienna, Austria.

[5] Core facilities, Medical University of Vienna, Vienna, Austria.

*Corresponding Author: gerald.prager@meduniwien.ac.at

Supplementary Information

Supplemental Table S1: Samples analysed retrospectively in this study. RNA-sequencing was performed on six patients with liver metastasis. DNA profiling was performed on 12 samples. Two samples were extracted from primary tumors and the remaining samples from metastases of different individuals, with one exception where samples originated from the same patient (highlighted in grey). References: OS: overall survival.

**RNA-seq (n=6)**			
**Short OS**		**Long OS**	
**Origin of tissue**	**OS**	**Origin of tissue**	**OS**
Liver	9 months	Liver	22 months
Liver	7 months	Liver	16 months
Liver	11 months	Liver	21 months
**DNA Cancer Panels (n=12)**			
**Short OS**		**Long OS**	
**Origin of tissue**	**OS**	**Origin of tissue**	**OS**
Liver	9 months	Liver	19 months
Liver	9 months	Liver	11 months
Liver	7 months	Lung	15 months
Pancreas	9 months	Lung	21 months
Pancreas	10 months	Liver	22 months
Liver	10 months	Liver	19 months

Supplemental Table S2: Genes covered by the Illumina TruSeq Amplicon Cancer panel.

ABL1	EGFR	GNAS	MLH1	RET
AKT1	ERBB2	HNF1A	MPL	SMAD4
ALK	ERBB4	HRAS	NOTCH1	SMARCB1
APC	FBXW7	IDH1	NPM1	SMO
ATM	FGFR1	JAK2	NRAS	SRC
BRAF	FGFR2	JAK3	PDGFRA	STK11
CDH1	FGFR3	KDR	PIK3CA	TP53
CDKN2A	FLT3	KIT	PTEN	VHL
CSF1R	GNA11	KRAS	PTPN11	
CTNNB1	GNAQ	MET	RB1	

Supplemental Table S3

**Sample ID**	**Source: https://genome-cancer.ucsc.edu/.**
TCGA-2J-AABT-01	
TCGA-F2-6880-01	
TCGA-HZ-8002-01	
TCGA-HZ-8003-01	
TCGA-HZ-A77P-01	
TCGA-IB-8126-01	
TCGA-IB-AAUQ-01	
TCGA-IB-AAUR-01	
TCGA-XD-AAUG-01	
TCGA-XD-AAUH-01	
TCGA-FB-A4P6-01	
TCGA-HZ-8315-01	
TCGA-LB-A8F3-01	
TCGA-OE-A75W-01	
TCGA-F2-7273-01	
TCGA-F2-7276-01	
TCGA-HZ-7918-01	
TCGA-HZ-7920-01	
TCGA-HZ-7923-01	
TCGA-HZ-8005-01	
TCGA-HZ-A77Q-01	
TCGA-IB-7893-01	
TCGA-IB-A6UG-01	
TCGA-IB-AAUS-01	
TCGA-IB-AAUT-01	
TCGA-RL-AAAS-01	
TCGA-2L-AAQI-01	
TCGA-2L-AAQL-01	
TCGA-FB-A78T-01	
TCGA-HZ-A49I-01	
TCGA-IB-A5SO-01	
TCGA-IB-AAUM-01	
TCGA-YB-A89D-01	
TCGA-F2-A7TX-01	
TCGA-IB-7646-01	
TCGA-IB-A5SQ-01	
TCGA-L1-A7W4-01	
TCGA-LB-A7SX-01	
TCGA-Z5-AAPL-01	
TCGA-2J-AAB8-01	
TCGA-2L-AAQA-01	
TCGA-3A-A9IZ-01	
TCGA-3A-A9J0-01	
TCGA-F2-6879-01	
TCGA-FB-AAPU-01	

TCGA-FB-AAQ1-01
TCGA-HZ-A4BH-01
TCGA-HZ-A77O-01
TCGA-IB-7886-01
TCGA-IB-AAUW-01
TCGA-YH-A8SY-01
TCGA-2J-AAB6-01
TCGA-2J-AABP-01
TCGA-3A-A9IB-01
TCGA-FB-A4P5-01
TCGA-FB-A545-01
TCGA-FB-A7DR-01
TCGA-H6-8124-01
TCGA-HZ-7922-01
TCGA-HZ-8317-01
TCGA-LB-A9Q5-01
TCGA-RB-AA9M-01
TCGA-XD-AAUI-01
TCGA-2J-AAB1-01
TCGA-2J-AABU-01
TCGA-2L-AAQJ-01
TCGA-F2-A44G-01
TCGA-FB-AAQ2-01
TCGA-FB-AAQ3-01
TCGA-FB-AAQ6-01
TCGA-H8-A6C1-01
TCGA-HZ-8638-01
TCGA-HZ-A8P1-01
TCGA-IB-7644-01
TCGA-IB-7887-01
TCGA-IB-A7LX-01
TCGA-IB-AAUN-01
TCGA-IB-AAUO-01
TCGA-IB-AAUU-01
TCGA-Q3-AA2A-01
TCGA-US-A77G-01
TCGA-2J-AAB9-01
TCGA-2J-AABR-01
TCGA-2L-AAQM-01
TCGA-3A-A9I7-01
TCGA-3A-A9IJ-01
TCGA-3A-A9IL-01
TCGA-3A-A9IN-01
TCGA-3A-A9IO-01
TCGA-3A-A9IR-01
TCGA-3A-A9IS-01
TCGA-3A-A9IV-01

TCGA-3A-A9IX-01
TCGA-F2-A44H-01
TCGA-HZ-8001-01
TCGA-HZ-8519-01
TCGA-HZ-A49G-01
TCGA-IB-7649-01
TCGA-IB-7888-01
TCGA-IB-8127-01
TCGA-Q3-A5QY-01
TCGA-2J-AABA-01
TCGA-2J-AABE-01
TCGA-2J-AABK-01
TCGA-2J-AABO-01
TCGA-2J-AABV-01
TCGA-3A-A9I9-01
TCGA-3A-A9IC-01
TCGA-H6-A45N-01
TCGA-HZ-8636-01
TCGA-IB-7889-01
TCGA-S4-A8RP-01
TCGA-IB-7645-01
TCGA-IB-7654-01
TCGA-IB-7890-01
TCGA-IB-A5ST-01
TCGA-IB-AAUV-01
TCGA-2J-AABH-01
TCGA-3A-A9IU-01
TCGA-FB-AAPQ-01
TCGA-FB-AAPY-01
TCGA-HZ-A49H-01
TCGA-IB-7652-01
TCGA-IB-A6UF-01
TCGA-PZ-A5RE-01
TCGA-RB-A7B8-01
TCGA-S4-A8RM-01
TCGA-US-A77E-01
TCGA-XD-AAUL-01
TCGA-2J-AABI-01
TCGA-3A-A9I5-01
TCGA-FB-A5VM-01
TCGA-HZ-7289-01
TCGA-HZ-7924-01
TCGA-IB-7647-01
TCGA-IB-7891-01
TCGA-IB-A5SS-01
TCGA-IB-A7M4-01
TCGA-IB-AAUP-01
TCGA-US-A774-01
TCGA-2L-AAQE-01

TCGA-3A-A9IH-01
TCGA-3E-AAAZ-01
TCGA-FB-AAPP-01
TCGA-HZ-7926-01
TCGA-HZ-A4BK-01
TCGA-US-A776-01
TCGA-HZ-7925-01
TCGA-IB-7651-01
TCGA-IB-7885-01
TCGA-IB-7897-01
TCGA-US-A77J-01
TCGA-2J-AAB4-01
TCGA-2J-AABF-01
TCGA-3E-AAAY-01
TCGA-F2-A8YN-01
TCGA-FB-AAQ0-01
TCGA-HZ-7919-01
TCGA-IB-A5SP-01
TCGA-M8-A5N4-01
TCGA-S4-A8RO-01
TCGA-US-A779-01
TCGA-YY-A8LH-01

Supplemental Figure S4

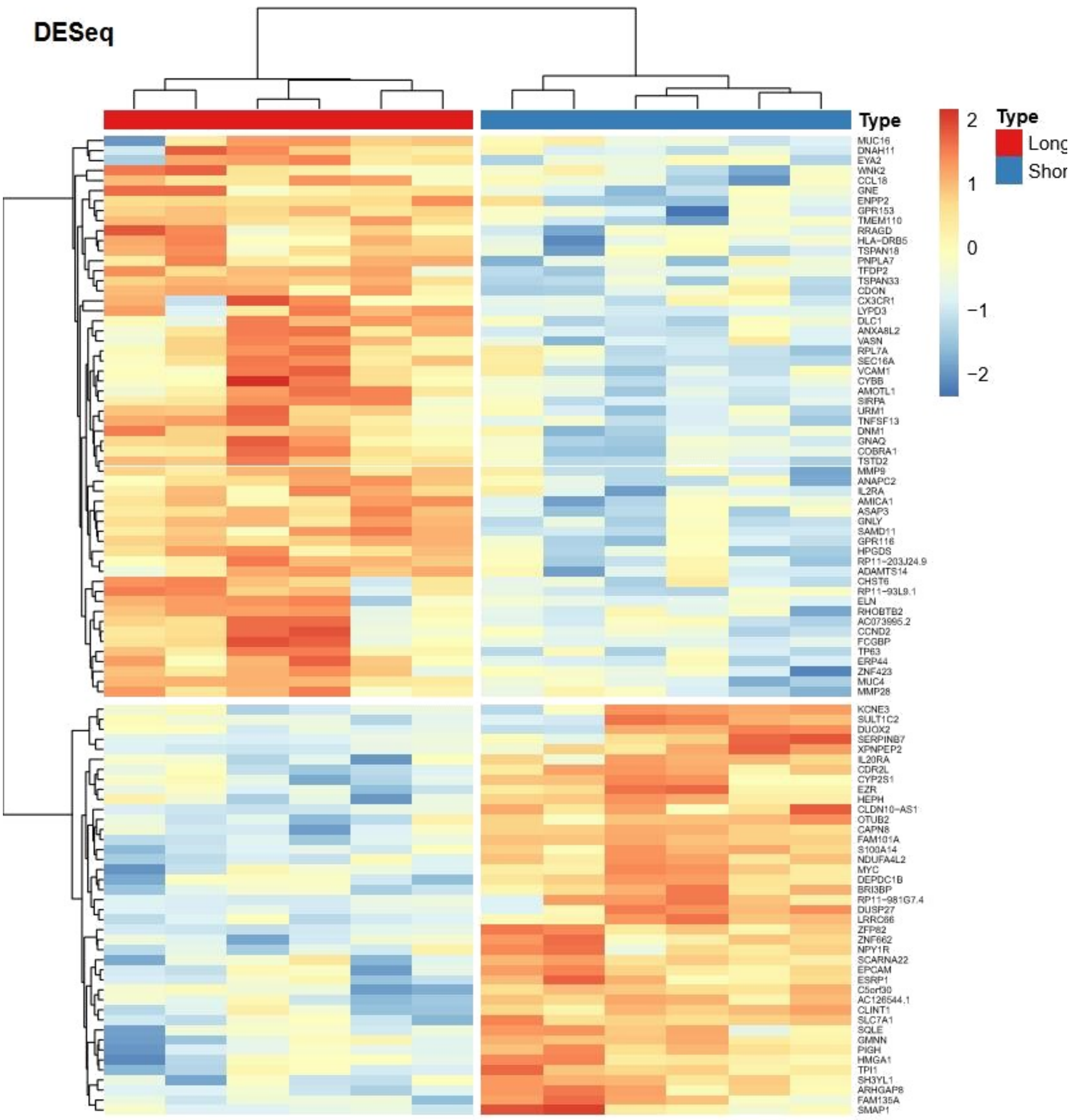

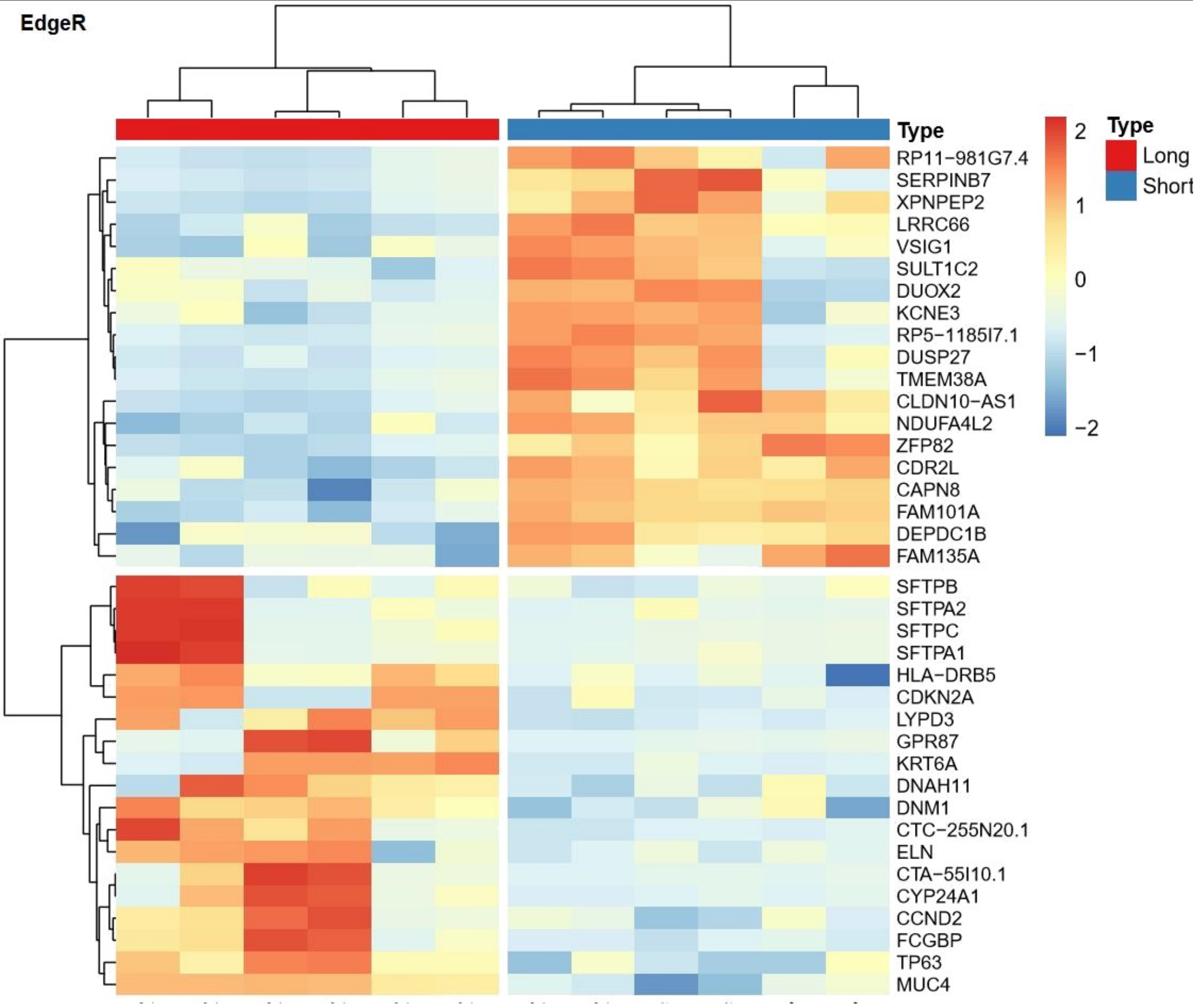
EdgeR
Type
Long
Short
2
1
0
−1
−2
RP11−981G7.4
SERPINB7
XPNPEP2
LRRC66
VSIG1
SULT1C2
DUOX2
KCNE3
RP5−1185I7.1
DUSP27
TMEM38A
CLDN10−AS1
NDUFA4L2
ZFP82
CDR2L
CAPN8
FAM101A
DEPDC1B
FAM135A
SFTPB
SFTPA2
SFTPC
SFTPA1
HLA−DRB5
CDKN2A
LYPD3
GPR87
KRT6A
DNAH11
DNM1
CTC−255N20.1
ELN
CTA−55I10.1
CYP24A1
CCND2
FCGBP
TP63
MUC4

Supplemental Table S5: Significance of genes on survival between survival type cohorts (short / long). Wilcox ranked sum test was performed to assess the significance of genes on survival duration between short and long survival cohorts before and after multiple testing correction using the Benjamini-Hochberg method.

Gene	Unadj. P-value	Adj. P. value
SMAD4	0,0080	0,1459
FLT3	0,0121	0,1459
NOTCH1	0,0183	0,1459
FBXW7	0,0194	0,1459
ATM	0,0200	0,1459
SMARCB1	0,0263	0,1459
ABL1	0,0289	0,1459
GNAQ	0,0295	0,1459
TP53	0,0295	0,1459
ERBB4	0,0298	0,1459
APC	0,0427	0,1570
PDGFRA	0,0431	0,1570
EGFR	0,0438	0,1570
STK11	0,0526	0,1570
GNA11	0,0542	0,1570
JAK3	0,0562	0,1570
KIT	0,0637	0,1570
FGFR3	0,0649	0,1570
AKT1	0,0698	0,1570
MLH1	0,0705	0,1570
ERBB2	0,0724	0,1570
IDH1	0,0731	0,1570
RET	0,0756	0,1570
PIK3CA	0,0771	0,1570
BRAF	0,0801	0,1570
KDR	0,0921	0,1736
VHL	0,1133	0,2057
PTEN	0,1262	0,2155
SMO	0,1275	0,2155
MET	0,1416	0,2250
FGFR2	0,1424	0,2250
NPM1	0,1620	0,2391
PTPN11	0,1629	0,2391
HRAS	0,1659	0,2391
CSF1R	0,1824	0,2535
HNF1A	0,1906	0,2535
FGFR1	0,1914	0,2535
ALK	0,2087	0,2691
RB1	0,2265	0,2846
SRC	0,2703	0,3312
CDH1	0,3629	0,4337
KRAS	0,4108	0,4793

EGFR,EGFR-AS1	0,4533	0,5165
JAK2	0,4898	0,5362
MPL	0,4924	0,5362
CDKN2A	0,5914	0,6299
CTNNB1	0,6746	0,7033
GNAS	0,7304	0,7456
NRAS	0,8693	0,8693
